# Influence of Organo-Sepiolite on the Morphological, Mechanical, and Rheological Properties of PP/ABS Blends

**DOI:** 10.3390/polym11091493

**Published:** 2019-09-12

**Authors:** Kui Wang, Tiantian Li, Sen Xie, Xianshun Wu, Weijiang Huang, Qin Tian, Chunyun Tu, Wei Yan

**Affiliations:** 1 School of Chemistry and Materials Engineering, Guiyang University, Guiyang 550005, China; 2 National Engineering Research Center for Compounding and Modification of Polymer Materials, Guiyang 550014, China

**Keywords:** polymer blends, sepiolite, mechanical property, crystallization, rheology

## Abstract

To improve the poor impact toughness of polypropylene (PP), organo-sepiolite (O-Sep) filled 80/20 (*w/w*) polypropylene/poly(acrylonitrile-butadiene-styrene) (PP/ABS) nanocomposites were fabricated. The contents of O-Sep were correlated with the morphological, mechanical, and rheological behavior of PP/ABS/O-Sep blends. Scanning electron microscopy (SEM) was applied to study the morphology and thermogravimetric analysis (TGA) was applied to study the thermal stability. Differential scanning calorimetry (DSC) and X-ray diffraction (XRD) were applied to study the crystallinity. The obtained results show that O-Sep enhanced the dispersion of ABS in the PP matrix and increased the crystallinity of blends. The rheological results show that O-Sep could increase the viscosity, storage modulus, and loss modulus of blends. Moreover, the mechanical behavior shows that O-Sep (at proper content) simultaneously increased the tensile modulus, flexural modulus, and impact strength of PP/ABS/O-Sep blends.

## 1. Introduction

Due to its low density, good elongation, high thermal stability, and good solvent resistance, polypropylene (PP) has become one of the most popular engineering plastics. However, low temperature brittleness and poor impact resistance restrict its applicability. Among all strategies for PP toughening, the introduction of a *β*-nucleating agent has been demonstrated to be the most reliable strategy [1,2,3,4,5,6,7,8]. Nowadays, due to the low cost, simple processing, and their non-toxic, macromolecular *β*-nucleating agents (such as polystyrene (PS), acrylonitrile-butadiene-styrene graft copolymer (ABS), or thermo-plastic phenolic resin) have been reported to have higher *β*-nucleating efficiency compared with low molecular weight organic *β*-nucleating agents. Moreover, benzene rings in the macromolecular structure may serve as a growth center for crystals [9,10,11,12,13,14,15,16,17,18]. Due to the advantages it offers, polymer blending technology has become one of the major strategies for the research and development of polymer materials in the past decade [19,20,21,22,23]. In a blend, the less viscous components form the continuous phase and the contents of dispersed phase determine the final morphology and thermo-mechanical properties. According to the mutual solubility and melt viscosity, polymers can be divided into compatible and incompatible polymers. Therefore, a physical or chemical modification of commodity is commonly applied in polymer blending technology.

ABS resin has a two-phase structure, has been demonstrated to perform like a typical rubber and improves the impact resistance of PP. ABS resin has also been reported to induce *β*-crystallization in the PP matrix [24,25,26,27]. PP is a crystalline, non-polar polymer, and ABS resin is a polar, random form polymer. The solubility parameters of both polymers are greatly different. Therefore, the interfacial tension between PP and ABS is large when blending, and additional improvement of the interface compatibility is necessary. At present, compatibilizer agent is one of the most popular strategies to improve the interface compatibility of polymeric blends.

Inorganic nanoparticles have been widely used as nanofillers to toughen polymers and their strong interfacial interaction and good dispersion are key attributes. The selective distribution of inorganic nanoparticles can regulate the morphology of the dispersed phase and further improve the compatibility of blends [28,29,30,31,32,33,34,35,36]. It has been reported that many nanoparticles, including SiO_2_, ZnO, CaCO_3_, carbon nanotubes, and montmorillonite, could induce *β*-nucleation in PP, which resulted in higher crystallization rates [37,38,39,40,41,42,43,44,45,46]. Due to the large specific surface, high surface activity, and lack of abrasion, sepiolite (Sep) is an ideal enhancer for polymers [47,48,49,50,51]. After the Sep nanofiber is de-bundled or modified, it can be compounded with polymers and form new materials with many excellent properties. A small amount of Sep nanofibers can greatly improve the performance of polymers. To enable Sep to be uniformly dispersed in polar or non-polar systems, Sep is usually surface-modified. To date, most research works have focused on the organic modified Sep (O-Sep) and have introduced O-Sep to a single polymer matrix to achieve a nanocomposite with excellent flame retardancy or mechanical properties. However, there are limited reports on the effects of O-Sep on the mechanical, thermal, and rheological properties of PP/ABS composite system.

In this paper, ABS is applied as special *β*-nucleating agent for PP toughening, and the potential role of Sep in the immiscible PP/ABS blends is investigated. To improve the interface compatibility between Sep and the tested polymers, Sep nanofibers are firstly modified with the silane coupling agent CG–570 to prepare organo-sepiolite (O-Sep). PP/ABS (80/20) blends containing various contents of O-Sep were prepared and the corresponding thermo-mechanical properties were investigated. Fourier transform infrared spectroscopy (FTIR) was applied to demonstrate the modification of Sep by the silane coupling agent CG–570. Scanning electron microscopy (SEM) was used to observe the morphology of PP/ABS and PP/ABS/O-Sep and the decentralization of ABS in the PP matrix. A TIRA 2700 machine was used to characterize the mechanical properties of PP/ABS/O-Sep. Thermogravimetric/derivative thermogravimetric analysis (TG/DTG) was used to investigate the thermal behavior of PP, PP/ABS, and PP/ABS/O-Sep. Differential scanning calorimetry (DSC) was used to study the crystallization of PP, PP/ABS, and PP/ABS/O-Sep. Rheological analysis was used to study the dynamic rheological properties of PP, PP/ABS, and PP/ABS/O-Sep.

## 2. Materials and Methods

### 2.1. Materials

Isotactic polypropylene (PP), grade T30S, with melt flow rate of 3 g/10 min, was purchased from Wuxi Kaihao Plastic Chemical Co., Ltd., Wuxi, China. Acrylonitrile-butadiene-styrene graft copolymer (ABS), grade PA–757, with specific gravity of 1.04 and melt flow rate of 1.8 g/10 min, was purchased from Taiwan Chimei Industrial Co., Ltd., Tainan, China. Fibrous type Sep (99%) was purchased from Sigma Aldrich and was dried at 100 °C for 8–10 h before processing. Silane coupler CG–570 (≥98%), with density about 1.070 g/cm^3^ and refractive index about 1.425 at 25 °C, was purchased from Nanjing Chengong Silicone Material Co., Ltd., Nanjing, China. All chemicals were used as received without further purification.

### 2.2. Modification of Sep

Sep was modified by surfactants via traditional strategy. Firstly, Sep powder was washed with deionized water three times and dried in an oven at 80 °C for 12 h. After that, the dried Sep was added to HCl/H_2_O (v/v 1:7) and stirred at room temperature for 2 h. After standing at room temperature for 24 h, the mixture was washed with deionized water 3–5 times, and then dried in an oven at 80 °C for 12 h to obtain acidified Sep. Secondly, the silane coupling agent CG–570 was added into ethanol/H_2_O (v/v 18:1) and the pH of the mixed solution was adjusted to 4–6 with formic acid. At last, CG–570 aqueous solution was added to the acidified Sep aqueous solution with 8 wt% content relative to acidified Sep, and stirred at 80 °C for 3 h. The final product was precipitated in ethanol by centrifugation at 5000 rpm for 3 min and dried at 60 °C for 24 h, then organo-sepiolite (O-Sep) was prepared.

### 2.3. Preparation of PP /ABS/O-Sep Blends

Polymers (PP and ABS) and O-Sep were dried at 80 °C for 4 h prior to mixing. The nanocomposites were prepared by a typical melt blending method at 180 °C with a rotation speed of 60 rpm for 8 min. Test specimens were prepared through an injection molding machine. The detail injection molding parameters were set as follow: melting temperature 180 °C, mold temperature 40 °C, hold pressure 0.7 MPa, and holding time 8 s. Table 1 shows the details of each sample composition and the corresponding sample designation.

### 2.4. Measurements and Characterizations

A FTIR spectrometer (Nicolet IS50, Thermo Fisher Scientific Inc., Waltham, MA, USA) was applied to characterize Sep, CG–570, and O-Sep, and the test resolution was 4 cm^−1^, the number of scans was 32, and the test range was 400–4000 cm^−1^. A FEI Quanta 250 FEG field-emission scanning electron micro-scope (SEM) (Thermo Fisher Scientific Inc., Waltham, MA, USA) under high vacuum at a voltage of 20 kV was applied to observe the morphologies of all testing samples, all testing samples were cryofractured with the direction parallel to the injection molding direction and coated with a thin layer of Pt. A TG 219 F3 thermal analyzer (Netzsch Instruments Co., Ltd., Hanau, Germany) was applied at a constant scanning rate of 10 °C/min under nitrogen at temperatures ranging from 50 to 600 °C to evaluate the thermal stability of all testing materials. DSC 214 (Netzsch Instruments, Co., Ltd., Hanau, Germany) at a heating rate of 10 °C/min was applied to study the crystallization of all testing samples. The samples were heated from 30 to 230 °C and held at this temperature for 5 min and then cooled to 30 °C at a rate of 10 °C/min. This was repeated to obtain a second heating scan. XRD (D/MAX2500, Rigaku Corporation, Tokyo, Japan) with Cu *Kα* radiation (*λ* = 1.54 Å) over the range of 5°–35° at a scanning rate of 2°/min was applied to study the crystalline structure of Sep and the blends, and the measurement geometry was Bragg-Brentano geometry, the voltage was 40 kV, the filament current was 40 mA. CMT6104 (MTS, Beijing, China) was applied to study the tensile performance testing (sample size: 75 mm × 5 mm × 1.88 mm) and bending performance testing (sample size: 64 mm × 10 mm × 4 mm), and ZBC1400-B (MTS, Beijing, China) was applied to study the impact performance testing (sample size: 64 mm × 10 mm × 4 mm). HAAKE MARS III (PolyLab OS; Thermo Fisher Scientific Inc., Waltham, MA, USA) was used to study the dynamic rheological properties of all testing samples.

## 3. Results and Discussions

### 3.1. Modification of Sep

**Figure 1:**
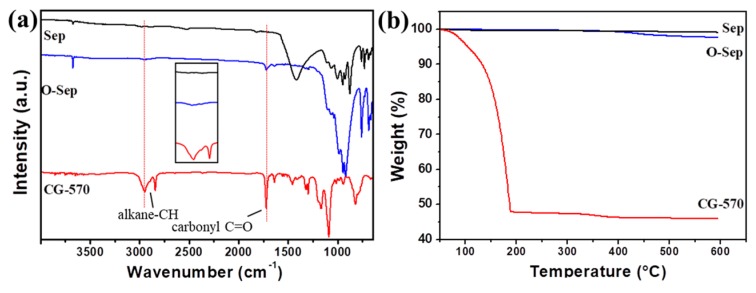
(**a**) FTIR and (**b**) TG curves of sepiolite (Sep), CG570, and organo-sepiolite (O-Sep), the dark lines, red lines, and blue lines in the figure represent Sep, CG–570, and O-Sep, respectively.

To improve the dispersion of Sep in the PP/ABS blends, Sep was modified with diluted HCl and CG–570 to prepare O-Sep. As shown in the FTIR curves in Figure 1a, O-Sep has an asymmetric stretching vibration peak of an alkane–CH bond at 2946 cm^−1^ [52] and a stretching vibration peak of carbonyl C=O at 1718 cm^−1^. The strong and wide absorption band at 1093 cm^−1^ shows the stretching vibration absorption peak of the Si–O–Si bond. The stretching vibration peak of Si–O bond at 910 cm^−1^ indicated that Si–OH reacted with Sep. FTIR demonstrated that CG570 successfully modified Sep. The further TG results (Figure 1b) were consistent with the FTIR results, and neither Sep nor O-Sep had hydroxyl water and coordination water (no obvious peak at 3500–3600 cm^−1^), Sep and O-Sep are very stable below 433.9 °C. After organic modification with CG–570, the mass loss of Sep increased, which further indicated that Sep has been modified with CG–570 successfully.

### 3.2. Morphology of Blends

**Figure 2:**
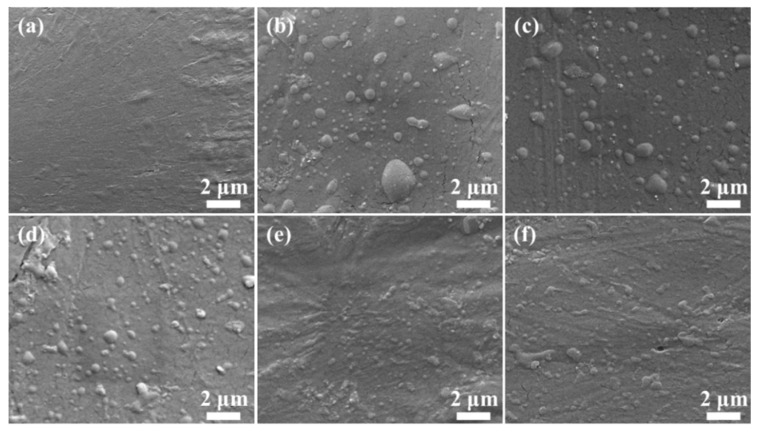
SEM images of cryofractured surfaces of PP and PP/ABS/O-Sep nanocomposites: (**a**) PP, (**b**) PP/ABS, (**c**) PP/ABS/O-Sep1, (**d**) PP/ABS/O-Sep3, (**e**) PP/ABS/O-Sep5, and (**f**) PP/ABS/O-Sep7.

A *β*-nucleating agent-based method has been demonstrated to be the most reliable strategy for PP toughening, and the dispersibility of the *β*-nucleating agent is key during this procedure. As shown in Figure 2a–f, the SEM images represent PP and PP/ABS/O-Sep with various O-Sep contents. For pure PP (Figure 2a), the SEM image shows homogenous phase and has no aggregation. For nanocomposite in the absence of O-Sep (Figure 2b), the SEM image shows that ABS microspheres dispersed in the PP matrix, showing a clear interface of both phases. In Figure 2c–f, the O-Sep is 1 wt%, 3 wt%, 5 wt%, and 7 wt%, respectively. When introducing O-Sep into PP/ABS blends, the aggregation of ABS in the PP matrix gradually decreased, and the interface of the two-phase gradually blurred. This indicates that the introduction of O-Sep into the blends improved the dispersion of ABS in the PP matrix. In summary, the phase morphologies in the SEM images demonstrate that O-Sep has good compatibility with PP/ABS blends and the introduced O-Sep may serve as a special compatibilizer for PP/ABS blends.

### 3.3. Mechanical Properties

To investigate the influences of *β*-nucleating and O-Sep on the mechanical properties of PP, the influences of ABS on the mechanical properties of PP were investigated first. Then, the influences of O-Sep on the mechanical properties of PP/ABS blends were investigated. Table 2 shows the results of the mechanical properties for pure PP, PP/ABS blends, and PP/ABS/O-Sep nanocomposites. This shows that addition of 20% ABS could improve the tensile strength and impact strength of PP. Moreover, tensile strength, blending strength, and impact strength increased when 1 wt% O-Sep was added to PP/ABS blends. Furthermore, in addition to the continuing increase of O-Sep content, all mechanical properties continued to increase when compared with PP/ABS blends. O-Sep has an optimum content for the tensile modulus, blending strength, blending modulus and impact strength of nanocomposite. As a comparison, other researchers have reported the modification of mechanical properties of PP. Bonda and co-workers have reported that the tensile strength of PP and PP/ABS (20%) is 29.0 and 31.8 MPa respectively [26]. In our experiments, the tensile strength of PP and PP/ABS (20%) is 36.3 and 38.6 MPa respectively. Liu and co-workers have demonstrated that ABS and nano-ZnO could toughen PP. For instance, the impact strength of PP and PP/ABS/ZnO is 3.31 and 10.29 kJ/m^2^ respectively in their experiments [24]. In our experiments, the impact strength of PP, PP/ABS, and PP/ABS/O-Sep5 is 4.54, 5.50, and 6.39 kJ/m^2^ respectively.

In short, adding O-Sep to the blends achieved better mechanical properties when compared with pure PP and PP/ABS blends. O-Sep may serve as a compatibilizer and help to improve interfacial adhesion, which is also supported by the results of SEM images shown in Figure 2. When sufficient O-Sep has been added, the dispersion of O-Sep into the PP/ABS matrix will become difficult, and the corresponding mechanical properties will begin to decrease [49].

### 3.4. Thermal Properties

#### 3.4.1. Thermogravimetric Analysis

**Figure 3:**
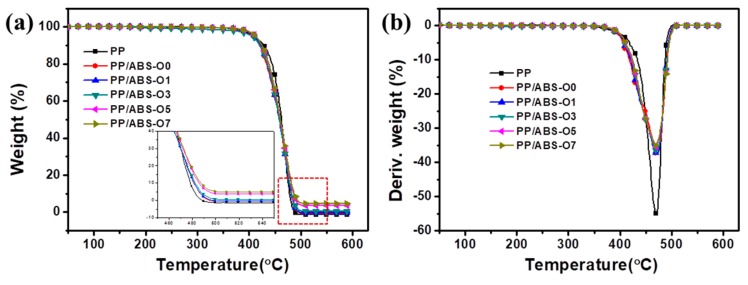
(**a**) TG and (**b**) DTG curves of PP and PP/ABS/O-Sep nanocomposites (heating rate 20 °C/min).

Figure 3a,b show the TG and DTG curves of PP and PP/ABS/O-Sep, respectively, and all curves show a typical one-step decomposition. Below 400 °C, almost no mass loss can be observed, demonstrating no physically bound water and crystallization water in the blends. This tendency was consistent with the TG curve of O-Sep in Figure 1b. Figure 3a,b show that when comparing PP with PP/ABS, the blending of ABS into the PP matrix could change the second step decomposition from 444–482 °C to 433–486 °C, and the *T*_max_ (the temperature of maximum mass loss rate) has no obvious change (PP: 470 °C; PP/ABS: 471 °C). Moreover, the curves of blends were almost overlapping and the final residual quality increased with increasing O-Sep in the blend. The mass loss of blends at the second step was between 430 and 486 °C, and *T*_max_ was about 471 °C. In summary, ABS and O-Sep have incorporated into the PP matrix and the introduction of ABS and O-Sep in these experiments almost did not affect the thermal stability of PP.

#### 3.4.2. Differential Scanning Calorimetry (DSC) and X-Ray Diffraction (XRD)

**Figure 4:**
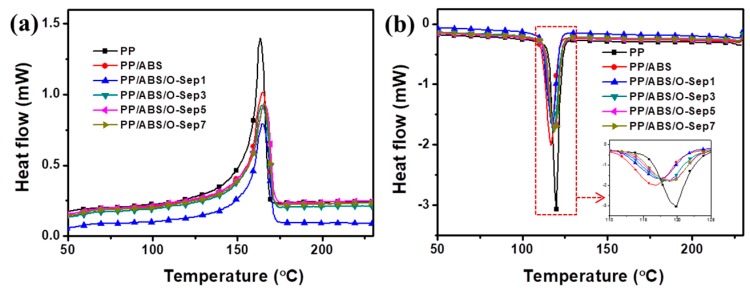
DSC traces of PP and PP/ABS/O-Sep nanocomposites: (**a**) second heating scans, (**b**) first cooling scans.

Figure 4a,b show DSC characterizations of pure PP and PP/ABS/O-Sep blends (second heating scans and first cooling scans), respectively. As shown in Figure 4a, the melting temperature of PP-based blends remained almost unchanged even after introducing ABS and O-Sep (neat PP: 163 °C; PP/ABS/O-Sep~165 °C). This indicates that the introduction of ABS and O-Sep did not affect the close packing of PP [49]. However, the introduction of ABS and O-Sep changed the crystallization temperature of PP (Figure 4b); 20 wt% ABS decreased the crystallization temperature of PP from 119 °C to 116 °C. When adding O-Sep to PP/ABS blends, the crystallization temperature of blends increased with increasing O-Sep (e.g., PP/ABS/O-Sep1: 116 °C; PP/ABS/O-Sep3: 118 °C; PP/ABS/O-Sep7: 119 °C). This indicates that O-Sep may act as a nucleating agent for the crystallization of PP/ABS, which was consistent with the conclusions of other researchers [41,48,53]. Since ABS and PP have relatively poor compatibility, introducing ABS into the PP matrix may decrease the crystallization ability of PP. O-Sep can improve the compatibility of PP and ABS and further increase the crystallization temperature of blends. This conclusion is also supported by the results of SEM images, and O-Sep can improve the interfacial adhesion between PP and ABS. Furthermore, XRD was applied to study the influences of ABS and O-Sep on the crystalline structure of PP.

**Figure 5:**
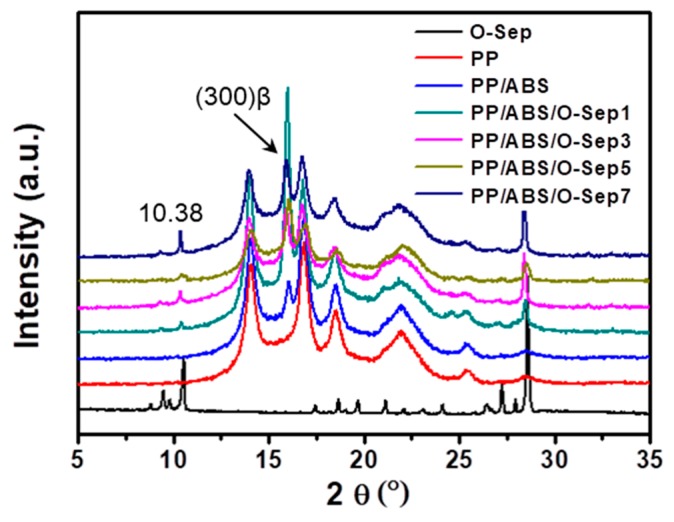
X-ray diffraction patterns of O-Sep, PP, PP/ABS and PP/ABS/O-Sep nanocomposites.

Figure 5 shows the XRD patterns of O-Sep and PP/ABS/O-Sep nanocomposites. The O-Sep has a peak at 2θ = 10.38 and the pure PP has five major peaks at 2θ between 5 and 30°. In PP/ABS/O-Sep nanocomposites, the peak intensity corresponding to 2θ = 10.38 increased along with the increase of O-Sep. Moreover, a clear peak was observed at about 16.06° for the PP/ABS/O-Sep blends, indicating that the *β*-crystal form existed in these nanocomposites [8,24]. The XRD result further confirmed that O-Sep could improve both the interfacial adhesion and the crystallization capacity of blends.

### 3.5. Rheological Properties

**Figure 6:**
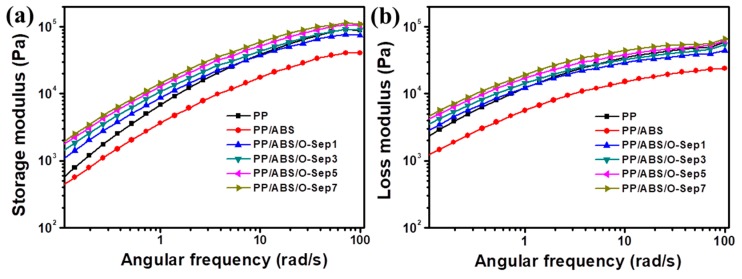
(**a**) Storage modulus and (**b**) loss modulus versus angular frequency for PP and PP/ABS/O-Sep nanocomposites at 210 °C.

The rheological behavior of polymers or polymer-based nanocomposites plays a vital role in their microstructure and processability [54,55]. It has been demonstrated that incorporating nanoparticles into a neat polymer could change the rheological properties of polymer matrix [56,57]. Figure 6a,b show the frequency dependence of the storage modulus (*G*’) and loss modulus (*G*″) for PP and PP/ABS/O-Sep. Due to the poor compatibility of ABS and PP, the introduction of ABS into PP matrix could induce a decrease of both storage modulus and loss modulus compared with neat PP. The curves of PP/ABS/O-Sep blends not only followed a monotonic increase with the content of O-Sep but also had almost the same enhancement of storage modulus and loss modulus at all frequencies. O-Sep improved the interface interaction of PP and ABS and the interaction among O-Sep nanoparticles could further induce apparent yield stress. O-Sep could form a network-like structure in the polymer matrix via silanol groups on their surface, and this special network-like structure could trap and immobilize polymer chains [58,59,60].

**Figure 7:**
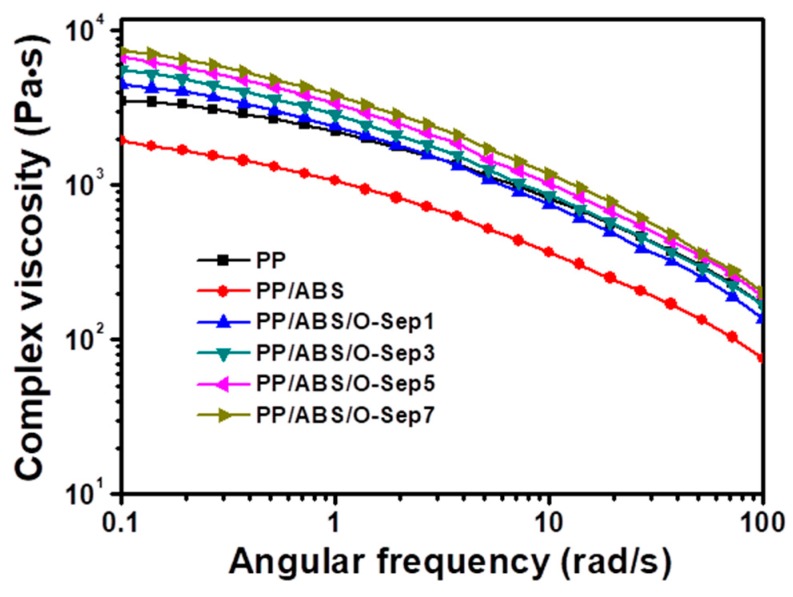
Plots of complex viscosity versus angular frequency of PP and PP/ABS/O-Sep nanocomposites at 210 °C.

The frequency dependence of the complex viscosity (η*) in Figure 7 shows that introduction of ABS decreased the complex viscosity of PP. Introduction of O-Sep generated a monotonic increase of complex viscosity of PP/ABS/O-Sep with increasing O-Sep in the low frequency region. The increase of complex viscosity of PP/ABS/O-Sep in low the frequency region indicated that O-Sep improved the interface interaction among O-Sep, ABS, and PP. However, the complex viscosity of PP/ABS/O-Sep blends gradually became similar with increasing frequency and final merged to one at higher frequency (>10 rad/s). This phenomenon may be induced by the high shear rate which ruptured the interface interaction among O-Sep, ABS, and PP.

## 4. Conclusions

In summary, PP/ABS/O-Sep nanocomposites (with various O-Sep contents) were prepared and the effects of O-Sep on the thermo-mechanical properties of nanocomposites have been systematically investigated. The results indicate that the toughness of PP can be improved by introducing both ABS and O-Sep. SEM images indicated improved dispersion of ABS in PP matrix by introducing O-Sep. The thermo-mechanical properties showed that not only the toughness of PP/ABS was enhanced by introducing O-Sep but also its crystallization temperature. Furthermore, the PP/ABS/O-Sep nanocomposites showed increased viscosity and modulus compared with PP/ABS blend and pure PP, and O-Sep acted as a reinforced filler. This work provides a new strategy to improve the PP/ABS blend system by introducing O-Sep as a nucleating agent and compatibilizer, and may provide a route for the toughening of PP.

## Figures and Tables

**Table 1:** Details of sample designations. Abbreviations: Polypropylene (PP), acrylonitrile-butadiene-styrene graft copolymer (ABS), and sepiolite (Sep).

Sample Designation	PP (g)	ABS (g)	O-Sep (g)
PP	60	—	—
PP/ABS	48.00	12.00	—
PP/ABS/O-Sep1	47.52	11.88	0.60
PP/ABS/O-Sep3	46.56	11.64	1.80
PP/ABS/O-Sep5	45.60	11.40	3.00
PP/ABS/O-Sep7	44.64	11.16	4.20

**Table 2:** Mechanical properties of PP and PP/ABS/O-Sep nanocomposites.

Sample	Tensile Strength (MPa)	Tensile Modulus (MPa)	Flexural Strength (MPa)	Flexural Modulus (MPa)	Impact Strength (kJ/m^2^)
PP	36.3 ± 0.6	1186 ± 17	42.6 ± 0.6	1839 ± 45	4.535 ± 0.233
PP/ABS	38.6 ± 0.5	1214 ± 21	41.4 ± 1.2	1723 ± 38	5.497 ± 0.285
PP/ABS/O-Sep1	40.8 ± 0.9	1229 ± 16	44.3 ± 1.5	1828 ± 42	5.834 ± 0.205
PP/ABS/O-Sep3	41.3 ± 0.8	1291 ± 14	47.2 ± 1.4	1739 ± 35	6.347 ± 0.267
PP/ABS/O-Sep5	42.0 ± 1.1	1331 ± 13	45.4 ± 0.8	1919 ± 47	6.392 ± 0.306
PP/ABS/O-Sep7	41.3 ± 0.9	1371 ± 23	45.8 ± 1.1	1612 ± 28	6.152 ± 0.254
